# Enriched Iron(III)-Reducing Bacterial Communities are Shaped by Carbon Substrate and Iron Oxide Mineralogy

**DOI:** 10.3389/fmicb.2012.00404

**Published:** 2012-12-03

**Authors:** Christopher J. Lentini, Scott D. Wankel, Colleen M. Hansel

**Affiliations:** ^1^School of Engineering and Applied Sciences, Harvard UniversityCambridge, MA, USA; ^2^Marine Chemistry and Geochemistry Department, Woods Hole Oceanographic InstitutionWoods Hole, MA, USA

**Keywords:** Fe, iron oxides, iron reduction, sulfate reduction, cultivation, niche differentiation

## Abstract

Iron (Fe) oxides exist in a spectrum of structures in the environment, with ferrihydrite widely considered the most bioavailable phase. Yet, ferrihydrite is unstable and rapidly transforms to more crystalline Fe(III) oxides (e.g., goethite, hematite), which are poorly reduced by model dissimilatory Fe(III)-reducing microorganisms. This begs the question, what processes and microbial groups are responsible for reduction of crystalline Fe(III) oxides within sedimentary environments? Further, how do changes in Fe mineralogy shape oxide-hosted microbial populations? To address these questions, we conducted a large-scale cultivation effort using various Fe(III) oxides (ferrihydrite, goethite, hematite) and carbon substrates (glucose, lactate, acetate) along a dilution gradient to enrich for microbial populations capable of reducing Fe oxides spanning a wide range of crystallinities and reduction potentials. While carbon source was the most important variable shaping community composition within Fe(III)-reducing enrichments, both Fe oxide type and sediment dilution also had a substantial influence. For instance, with acetate as the carbon source, only ferrihydrite enrichments displayed a significant amount of Fe(III) reduction and the well-known dissimilatory metal reducer *Geobacter* sp. was the dominant organism enriched. In contrast, when glucose and lactate were provided, all three Fe oxides were reduced and reduction coincided with the presence of fermentative (e.g., *Enterobacter* spp.) and sulfate-reducing bacteria (e.g., *Desulfovibrio* spp.). Thus, changes in Fe oxide structure and resource availability may shift Fe(III)-reducing communities between dominantly metal-respiring to fermenting and/or sulfate-reducing organisms which are capable of reducing more recalcitrant Fe phases. These findings highlight the need for further targeted investigations into the composition and activity of speciation-directed metal-reducing populations within natural environments.

## Introduction

Iron (Fe) (hydr)oxide minerals are potent repositories of nutrients (e.g., phosphate) and metal(loid)s (e.g., arsenic). Release of these elements into the aqueous milieu may occur upon dissolution of the Fe (hydr)oxides mediated by a number of abiotic and biotic processes. Under anaerobic conditions, reductive dissolution is primarily attributed to reaction with sulfide in sulfidogenic environments and microbial activity in non-sulfidogenic environments (Lovley et al., 1991). The biotic mechanisms of Fe(III) reduction within soils and sediments are primarily attributed to either an indirect consequence of fermentation or microbial respiration, in which organisms couple the oxidation of carbon or molecular hydrogen to the reduction of Fe(III) for energy conservation (dissimilatory reduction). Given the ubiquity of Fe oxides within soils and sedimentary systems, microbial Fe(III) reduction can have a profound impact on carbon cycling and degradation. In fact, microbial Fe(III) reduction accounts for a up to 50% of carbon oxidation in non-sulfidogenic sediments (Canfield et al., 1993; Thamdrup, 2000).

Most oxidized Fe within soils and sediments (pH > 4) exists as a variety of oxy(hydr)oxides (hereinafter referred to as oxides), where the three most common are ferrihydrite (Fe_5_HO_8_·4H_2_O), goethite (α-FeOOH), and hematite (α-Fe_2_O_3_). These Fe(III) phases vary greatly in their physiochemical properties, including solubility, reduction potential, and surface area. Ferrihydrite, the least crystalline and most soluble phase, supports the greatest extent and highest rates of Fe(III) reduction in laboratory incubations with common dissimilatory Fe(III)-reducing microorganisms (DIRMs; Lovley and Phillips, 1988; Roden, 2003; Hansel et al., 2004). In contrast, under identical conditions, goethite and hematite, phases with higher crystallinities and lower solubilities, support only minimal Fe(III) reduction (e.g., ∼1–5% of total Fe; Hansel et al., 2004) by these same organisms (Lovley and Phillips, 1988; Roden and Zachara, 1996; Fredrickson et al., 2003). Based on these laboratory observations, the availability of Fe(III) oxides for microbial respiration is believed to decrease in the order ferrihydrite to goethite to hematite.

Although ferrihydrite is believed to be the most bioavailable Fe(III) oxide, it is a transient phase having a short residence time in sediments where it ripens to more crystalline oxides such as goethite and hematite (Benner et al., 2002; Hansel et al., 2003). Furthermore, as a consequence of the high reactivity of ferrihydrite (Cornell and Schwertmann, 1996), its reduction potential and bioavailability is oftentimes compromised by extensive incorporation of other cations (Ekstrom et al., 2010; Hansel et al., 2011). Thus, the role of ferrihydrite in sustaining long-term DIRM populations and maintaining Fe(II) generation in mature sediments is likely minor (Hansel et al., 2004). In fact, in aged soils and sediments crystalline Fe(III) oxides are 2–10 times greater when compared to their less crystalline counterpart (Roden and Urrutia, 2002). Furthermore, it has been shown that crystalline Fe(III) oxides are preferentially reduced and responsible for the oxidation of carbon within some natural sediments (Stucki et al., 2007). Given the limited ability of model DIRM to reduce more crystalline Fe(III) oxides, a link between the reduction of these crystalline phases and microbial Fe(III) respiration (i.e., dissimilatory Fe(III) reduction) within the environment is questionable – or, at least not a consequence of dissimilatory reduction by well-characterized model DIRM (e.g., *Shewanella* spp., *Geobacter* spp.). This begs the question, what processes and microbial groups are responsible for the reduction of crystalline Fe(III) oxides within soil and sedimentary environments? Are there undiscovered dissimilatory Fe(III)-reducing microbes that have a better ability to reduce more crystalline (lower reduction potential) Fe(III) oxides? Or are other microbial processes responsible for Fe(III) reduction in more mature sedimentary systems?

Addressing these questions is complicated by the lack of a universal functional gene for Fe(III) respiration. Thus, here we take a first step at addressing these questions by identifying the microbial communities hosted within sediment enrichments containing Fe(III) oxides spanning a range of crystallinities. To do this, we employ the first large-scale cultivation effort using various Fe(III) oxides (ferrihydrite, goethite, hematite) and carbon sources (glucose, lactate, acetate) along a dilution gradient (10^−1^ to 10^−5^) to provide various ecological niches for Fe(III)-reducing communities. The tested carbon and Fe oxide sources span environments ranging in geochemical maturity and extent of carbon degradation. Further, enrichments along a dilution gradient target organisms occupying different ecological niches, in particular spanning life history traits (e.g., r and K strategies). The results observed here point to microbial groups and processes that are presumed responsible for Fe(III) reduction within contrasting geochemical environments and will therefore aid in the interpretation of Fe dynamics observed in field and natural sediment incubations.

## Materials and Methods

### Site description and sampling

Sediment was collected from Ashumet Pond, Falmouth, MA, USA, in August of 2008. Ashumet Pond is a freshwater kettle-hole pond that receives phosphate-laden groundwater from infiltration beds at a decommissioned wastewater treatment and disposal facility at the adjacent Massachusetts Military Reservation on Cape Cod. In 2004, a permeable reactive barrier containing zero valent Fe [3% Fe(0) by weight] was emplaced subhorizontally on the bottom of the pond (0.6 m deep) to intercept phosphate-laden water that discharges near the shore of Ashumet Pond (McCobb et al., 2009). Three shallow sediment cores were collected within the pond downgradient of the barrier with a water depth of ∼0.5 m. Upon sampling, the cores were stored on ice, transferred back to the laboratory and placed in an anaerobic chamber (94% N_2_: 6% H_2_). The outer 1 cm of the cores was discarded, the remaining sediment was combined and mixed, and 10 g of the homogenized sediment was placed in anaerobic serum bottles containing sterile HEPES buffer (10 mM). Sediments were thoroughly mixed before 10-fold serial dilutions were carried out in sterile 10 mM HEPES buffer to a final dilution of 10^−5^. These sediment slurries served as the inocula for the Fe(III) reduction enrichments.

### Mineral synthesis

Two-line ferrihydrite, goethite, and hematite were synthesized following the methods of Schwertmann and Cornell (2007) and characterized in detail previously (Hansel et al., 2003, 2004). All minerals were washed via repeated centrifugation, dialyzed in Spectra/Pur cellulose membrane tubing (MWCO 12,000–14,000) until a steady conductivity was obtained, and maintained as slurries (minimizing alteration). Following dialysis, the pH of mineral slurries was adjusted to 7.5 and stored at 4°C. For anaerobic enrichments, slurries were bubbled with N_2_, sealed in anaerobic serum bottles, and sterilized (10 min, 120°C). X-ray diffraction was conducted on samples before and after sterilization (Scintag XDS2000) to confirm Fe oxide identity and purity.

### Fe(III)-reducing enrichments

Enrichments were conducted in an anaerobic freshwater medium (Widdel and Bak, 1992), containing (in mM): MgSO_4_, 0.2; MgCl_2_, 4; CaCl_2_, 0.9; KH_2_PO_4_, 4.4; NH_4_Cl, 5.6; NaHCO_3_, 30, 1 mL L^−1^ trace metal solution, and 0.1 mL L^−1^ vitamin stock (pH 7.3). Media was dispensed into anaerobic Balch tubes (BellCo Glass) and the headspace exchanged with 80% N_2_/20% CO_2_ gas mixture. Prior to inoculation with sediment, media was amended with carbon (acetate, lactate, glucose, or a mixture of all three) to a final concentration of 10 mM and an Fe(III) oxide (ferrihydrite, goethite, or hematite) to a final total Fe concentration of 25 mM. Sediment suspensions along the dilution gradient (10^−1^ to 10^−5^) were added to a final inoculum of 10%. Enrichments were stored at room temperature in the dark and transferred every 3 months. Following growth of the third successive transfer, the enrichments were analyzed for Fe(II), organic carbon, and microbial community composition.

All enrichments were conducted in duplicate. Trends for Fe(III) reduction and enriched microbial communities were similar between duplicate enrichments. In some cases, however, the results were offset in dilution, likely a result of slight deviation in the initial inoculum added to the first dilution and the stochastic nature of microbial enrichments. For consistency and clarity, only one enrichment data set is presented.

### Iron chemistry of enrichments

Acid extracts were obtained using 1 mL of well-mixed enrichments added to 5 mL of concentrated HCl and shaken for 12 h to ensure complete dissolution of Fe phases. From this extract, total Fe(II) (dissolved and solid associated) was measured using the Ferrozine assay (Stookey, 1970). Within the same extract, total Fe was measured by first reducing all Fe(III) to Fe(II) with hydroxylamine hydrochoride (0.4 M, heated to near boiling).

### DNA extraction

DNA from enrichments was extracted from enrichment cultures using a modified protocol for the Ultraclean soil DNA kit (MoBio Laboratories). Enrichment cultures were centrifuged at 5000×*g* for 5 min. Supernatant was decanted off and the pellet was resuspended in 500 μL of nuclease free water. Prior to adding the bead beating solution, 200 μg of polyadenylic acid was added (Webster et al., 2003; Santelli et al., 2008).

### Terminal restriction fragment length polymorphism analysis

Terminal Restriction Fragment Length Polymorphism (T-RFLP) profiles were generated for all enrichment cultures. DNA was amplified with a 5′ fluorescently labeled forward primer (8F with 5-hexachlorofluorescein; HEX) and a 5′ fluorescently labeled reverse primer (1492R with 6-carboxyfluorescein; FAM). Two independent 50 μL PCRs were combined and purified using QIAquick nucleotide removal kit (Qiagen). The concentration of combined/purified DNA was determined via nanodrop and concentrated samples were diluted with nuclease free water to ensure proper digestion. Approximately 160 ng of purified DNA was digested in 20 μL reaction with 20 U of *MSPI*, *Hae*III, or *Hha*I for 4 h at 37°C. Duplicate T-RF profiles were prepared for electrophoretic analysis by adding 0.5 μL of digested products to 0.5 μL GeneScan -500 ROX size standard and 9.0 μL of Hi-Di Formamide. The loading mix was heated for 3 min at 95°C and immediately chilled on ice before analysis on a 3730xl ABI capillary sequencer.

### Cloning, sequencing, and phylogenetic analysis

A subset of samples were cloned to assign T-RFLP fragments to bacterial species. Amplification of the 16S rRNA gene was performed using the 8F and 1492R primer set (Lane, 1991) and conditions used previously (Hansel et al., 2008). Sequencing of PCR products were performed using the forward T3 and reverse T7 primers on an ABI 3730xl capillary sequencer. A total of 576 clones were sequenced to assign identity to 53 *Msp*I T-RFLP fragments. Sequencher 4.8 (Gene Codes, Corp.) was used to trim the cloning vector and poor quality reads from sequences before aligning the contigs and exporting consensus sequences for further analysis. Alignment of sequences was performed in ARB (Ludwig et al., 2004). Upon alignment of sequences, estimation of maximum likelihood phylogenies was performed using the online web interface of PhyML (Guindon et al., 2005). Robustness of clusters were tested with bootstrap resampling (*n* = 1000).

### Nucleotide sequence accession numbers

Sequences obtained in this study have been deposited in the GenBank database under accession numbers JX828409–JX828432 (bacterial 16S rRNA clones).

### Statistical analysis

Data was first visually analyzed to ensure quality electropherograms and then aligned and converted into data tables using Genemappers Local Southern method (Applied Biosystems). Peaks from 50 to 550 base pairs were exported for further analysis. The statistical analysis method implemented by Abdo et al. (2006) was used to discriminate between signal/noise and to align peaks into appropriate bins. In addition, duplicate digests were performed for each enrichment and peaks not present in both digests were discarded and the peak areas averaged. This method corrects for any potential differences in the amount of DNA injected between digest by relativizing peak area within each digest. T-RFs were then manually assigned to bacterial groups only if six out of six peaks matched the *in silico* digestion of phylotypes from clone libraries. Bulk phylogenetic groupings were obtained by combining similar organisms, for instance *Desulfovibrio putealis* (454 bp for *Msp*I) and *Desulfovibrio vulgaris* (286 bp for *Msp*I) were combined to produce one *Desulfovibrio* bin. In order to describe the pairwise dissimilarities between enrichments Bray–Curtis index was implemented from the vegdist function in the vegan package (Oksanen et al., 2012) of R^1^. Hierarchical cluster analysis on the set of dissimilarities was performed using the average agglomeration method (hclust function in the stats package of R) and annotated heatmaps were generated using the heatplus package in R (Ploner, 2011). Finally, the cophenetic correlation was calculated to insure the proper clustering of data when compared to pairwise dissimilarities.

## Results

### Fe(III) reduction in enrichment cultures

The amount of Fe(II) produced within sediment incubations varied as a function of carbon source, Fe(III) oxide type, and sediment dilution. Of the original 120 enrichments (40 for each mineral), 45 enrichments demonstrated reduction greater than 10% of the total Fe provided. The number of enrichments supporting Fe(III) reduction followed the predicted trend of Fe(III) bioavailability for the Fe oxides, with the majority of the Fe(III)-reducing enrichments obtained on ferrihydrite (60%) and goethite (36%), while hematite (4%) represented fewer. The effect of carbon source on these enrichments was also clear, with the most energetic carbon sources, mixed carbon (36%), glucose (33%), and lactate (22%) representing the majority of Fe(III)-reducing enrichments, while those enriched on acetate represented only 9%.

**Figure 1:**
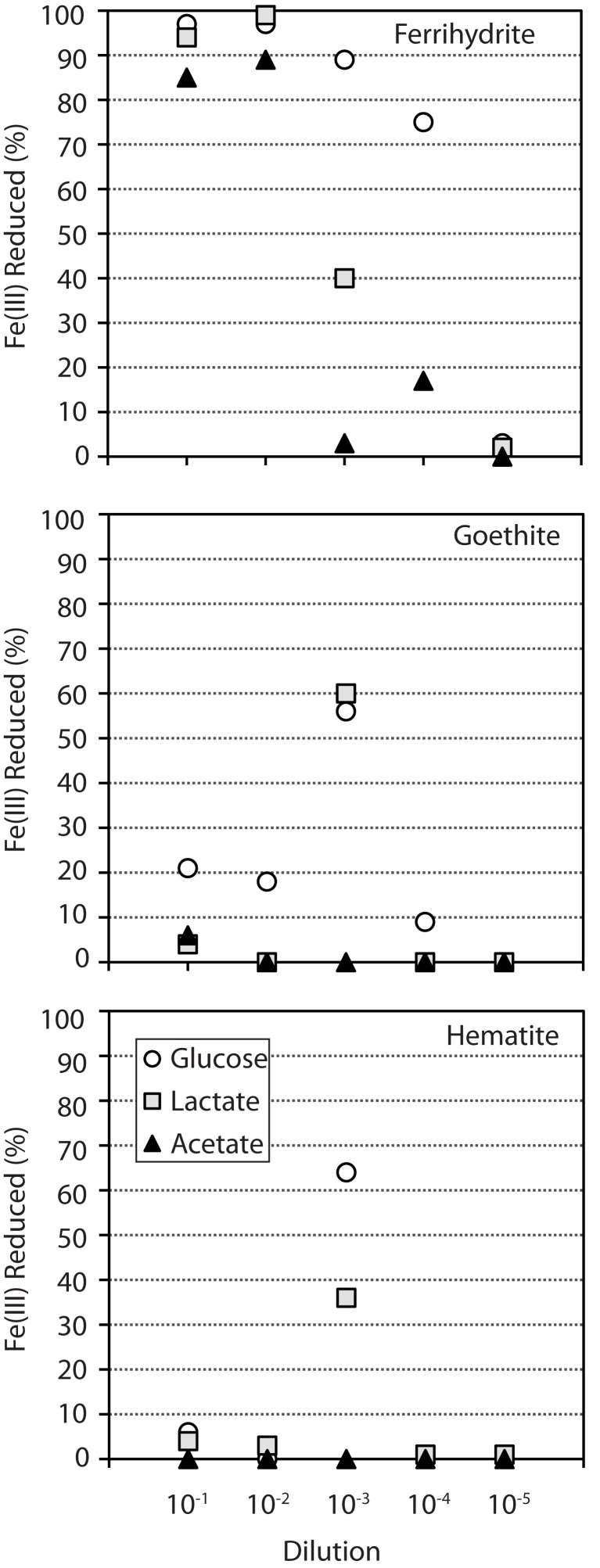
**Percent of total Fe(III) reduced (Fe^2+^/Fe_TOT_) in enrichments supplemented with different Fe oxide (each graph) and carbon sources (▲ = acetate ■ = lactate ● = glucose)**. The mixed carbon source enrichments, which behaved similarly to glucose, were omitted for clarity. Fe(III) reduction values for glucose enrichments with ferrihydrite (10^−5^), goethite (10^−5^), and hematite (10^−2^, 10^−4^, 10^−5^) are all 0 – the data point (circle) is not observed behind the lactate data point (square). Enrichments contained 25 mM Fe_Tot_ and an excess of carbon substrate (10 mM).

In general, for ferrihydrite, the trend for Fe(III) reduction was similar among all carbon compounds, with the percent of Fe(III) reduced decreasing progressively from the 10^−1^ dilution to 10^−5^ dilution (Figure 1). In contrast, within both goethite and hematite enrichments, the amount of Fe(II) produced showed a strong dependence on sediment dilution, with most reduction occurring in the 10^−3^ dilution culture when lactate and glucose were provided as carbon sources (Figure 1). Goethite also supported moderate (18–21%) Fe(III) reduction in the 10^−1^ and 10^−2^ dilutions for glucose. In contrast to ferrihydrite, no or minimal Fe(III) reduction was observed within goethite and hematite enrichments when acetate was provided as the carbon source.

### Terminal restriction fragment length polymorphism analysis

In order to further understand the microbial communities associated with the reduction of the various Fe(III) phases, only the 45 enrichments in which greater than 10% of the total Fe(III) was reduced were selected for further analysis. Preliminary T-RFLP analysis revealed low richness within these enrichment cultures (e.g., number of peaks ranging from 2 to 16), which allowed for a large-scale analysis of the 45 enrichments using T-RFLP analysis in combination with cloning for peak identification. The 16S rRNA gene was amplified with fluorescent forward and reverse primers and three separate restriction digests (*Msp*I, *Hae*III, *Hha*I) were performed to produce six distinct electrophoretograms for each enrichment culture. In combination with *in silico* digestion of known phylotypes from 16S rRNA gene clone libraries, T-RFs could be identified and matched to peaks in each electrophoretogram (see Table 1). In total, the *Msp*I enzyme yielded 73 distinct T-RFs in the 45 enrichment cultures, 24 T-RFs (33%) could be assigned to known bacterial groups while 49 (67%) peaks remain unidentified. Of the unidentified fraction, however, 44 were in 2 or fewer enrichments and consisted of relatively minor peaks, with the median unknown fraction being 7%. Within the 73 distinct T-RFs, a total of 252 peaks were detected with 62% of the peaks represented by identified clones. Seven major peaks constituted 51% of the detected peaks and made up 74% of the total peak area. These peaks corresponded to organisms with closely related organisms at the family-level, including Spirochaetaceae, Aeromonadaceae, Desulfovibrionaceae, Geobacteraceae, and the two families Eubacteriaceae and Clostridiaceae within the Gram positive division Firmicutes. The other dominant peaks did not have closely related cultured representatives at the family-level, which included organisms within the phylum Actinobacteria and a number of unclassified organisms within the phylum Bacteroidetes (Table 1). For the remainder of the manuscript, organisms constituting the seven most abundant peaks will be referred at the lowest and most informative identified taxonomic level (Spirochaetes, Actinobacteria, Firmicutes, Aeromonas, Bacteroidetes, Desulfovibrio, Geobacter). The remaining known peaks constituted another 7% of the total peak area.

**Table 1:** **Bacterial 16S rRNA phylogeny of sequenced clones with their closest cultured representative (NCBI)**.

	Forward			Reverse			Accession number	Top BLAST Hit and closest culturable representative (NCBI)		Designation in Tree (Figure 5)
	MspI	*Hha*I	*Hae*III	MspI	*Hha*I	*Hae*III				
**ACIDOBACTERIA**										
*Geothrix*										
FA4A, FA4B, FL1B, FL3B, FL4A*, GL3B, HL3A	278	359	201	92	380	125	HM141900	Uncultured bacterium clone MA-9-V94B 16S ribosomal RNA gene	98%	AP-FeEnrich1
							NR_036779	*Geothrix fermentans strain H5* 16S ribosomal RNA, partial sequence	98%	
**ACTINOBACTERIA**										
*Coriobacteriaceae*										
FL3A*, FG1A, FG2A, FG2B, FG3A*, FG3B, FG4A*, FG4B, FM1A, FM1B, FM2A, FM2B, FM3A, FM3B, FM4A, FM4B, GL3A*, GL3B, GG2B, GM3A, GM3B, GM4A, GM4B, HG3B, HL3A	172	284	229	128	45	69	GQ396959 EU592964	Uncultured bacterium clone AK1DE1_01E 16S ribosomal RNA gene *Olsenella* sp.*F0004* 16S ribosomal RNA gene	98% 89%	AP-FeEnrich2
**BACTEROIDETES**										
FG1A*, HG3B	90	94	NC	83	49	116	FJ269054.1	Iron-reducing bacterium enrichment culture clone HN19 16S ribosomal RNA gene	99%	AP-FeEnrich3
							AB214329.1	*Bacteroides intestinalis* gene for 16S rRNA, partial sequence, strain:JCM 13266	92%	
FL1A, FL2A*, FG1B, FG2A*, FM1A, FM1B, FM2A, GG1B	424	86	<50	33	47	112	EU662649	Uncultured bacterium clone MC1_16S_80 16S ribosomal RNA gene	98%	AP-FeEnrich4
							AB611036	*Bacteroidetes bacterium RL-C* gene for 16S rRNA, partial sequence	96%	
FG2A*, FM1A, GL3B, GM1B, GM2B	201	86	<50	33	47	112	AB447707.1	Uncultured bacterium gene 16S rRNA, partial sequence, clone: D242_27F_BAC_042	98%	AP-FeEnrich5
							AB611036.1	Bacteroidetes bacterium RL-C gene for 16S rRNA, partial sequence	96%	
HG3A*	95	100	<50	120	135	116	JQ617816.1	Uncultured Bacteroidetes bacterium clone Rc333 16S ribosomal RNA gene	96%	AP-FeEnrich6
							AB548674.1	*Dysgonomonas capnocytophagoides* gene for 16S ribosomal RNA, strin: JCM 16697	94%	
**CHLOROFLEXI**										
FL2A, GM3B, HG3B*	159	536	220	76	390	125	JN397974	Uncultured bacterium clone SSB0301-14 16S ribosomal RNA gene, partial sequence	99%	AP-FeEnrich7
							NR_040972	*Levilinea saccharolytica strain KIBI-1* 16S ribosomal RNA, partial sequence	94%	
**FIRMICUTES**										
*Clostridiales*										
*Clostridiaceae*										
*Clostridium*										
FG1A*, GM1A, GM1B, GM2A	179	193	271	124	403	43	GU370098	*Clostridium* sp.*P530(3)* 16S ribosomal RNA gene, partial sequence	98%	AP-FeEnrich8
*Eubacteriaceae*										
*Acetobacterium*										
FA1B, FL1A, FL2A, FL2B, FG1A, FG2A, FG2B, FG3B, FM1B, FM2A*, FM4B, GG1A, GG1B, GG2A*, GM1B, GM2A, GM2B	216	371	NC	127	401	NC	JX223412.1	Uncultured bacterium clone EMIRGE_OTU_s5t4a_1110 16S	99%	AP-FeEnrich9
							NR_026326	*Acetobacterium malicum strain DSM 4132* 16S ribosomal RNA > emb|X96957.1|	99%	
**β-PROTEOBACTERIA**										
*Burkholderia*										
HL3A*	492	564	200	125	51	255	FJ517671.1	Uncultured Burkholderiales bacterium clone 0-1_46 16S ribosomal RNA gene	99%	AP-FeEnrich10
							EU921644	*Duganella* sp.*LT1-9* 16S ribosomal RNA gene, partial sequence	99%	
**δ-PROTEOBACTERIA**										
*Syntrophaceae*										
FG1A*	210	95	197	73	403	125	JN038811	Uncultured *delta proteobacterium* clone P-R23 16S ribosomal RNA gene	99%	AP-FeEnrich11
							CP002629	*Desulfobacca acetoxidans* DSM 11109, complete genome	88%	
*Desulfobacter*
FA4A, FA4B	515	95	209	127	405	125	DQ831538	Uncultured *delta proteobacterium* clone WB03 16S ribosomal RNA gene	98%	AP-FeEnrich12
							AF328857	*Olavius algarvensis* sulfate-reducing endosymbiont 16S ribosomal RNA gene	94%	
*Desulfomicrobium*										
FA4A*, FG1B	161	90.5	71.2	65	46	124	AY604056.1	Uncultured bacterium clone DR9IPCB16SCT4 16S rRNA gene	99%	AP-FeEnrich13
							CP001629.1	*Desulfomicrobium baculatum* DSM 4028, complete genome	99%	
*Desulfovibrio*										
FA2B, FA4B, FL1A, FL1B, FL2A*, FL3A*, FG4B, FM2B, GL3A*, GL3B, HL3A*	457	95	201	127	142	123	NR_029118	*Desulfovibrio putealis strain B7-43* 16S ribosomal RNA > gb|AY574979.1|	99%	AP-FeEnrich14
FL3B, FL4A*	507	93	201	68	143	122	DQ205193	Uncultured *delta proteobacterium* clone MBNTA-bac1 16S small subunit RNA gene	97%	AP-FeEnrich15
							NR_029118	*Desulfovibrio putealis strain B7-43* 16S ribosomal RNA, > gb|AY574979.1|	97%	
FL1A, GL3A*	289	57	75	74	270	35	AY928231.1	Bacterium S9552 16S ribosomal RNA gene, partial sequence	99%	AP-FeEnrich16
							AY362360	*Desulfovibrio vulgaris strain I5* 16S ribosomal RNA gene, partial sequence	95%	
*Geobacter*										
FA1B, FA2B, FA4A*, FA4B, FL1B, FL2A, FL2B*, FG1A, FG2B	164	93	217	126	405	124	JN038618	Uncultured *delta proteobacterium* clone MA-R44 16S ribosomal RNA gene	98%	AP-FeEnrich17
							NR_026077	*Geobacter pelophilus* strain Dfr2 16S ribosomal RNA, > gb|U96918.1|	97%	
**γ-PROTEOBACTERIA**										
*Aeromonas*										
FA4A*, FA4B, FL1B, FG3A*, FG3B, FG4A*, FM2B, FM3A, FM3B, FM4A, GL3A*, GG2B, GM3A, GM3B, GM4A, GM4B, HG3B, HL3A*	90	215	<50	123	221	124	AY910844	*Aeromonas salmonicida* subsp.*achromogenes* 16S ribosomal RNA gene	99%	AP-FeEnrich18
	90	215	<50	123	221	124	FJ940821	*Aeromonas jandaei strain LNC206* 16S ribosomal RNA gene	99%	AP-FeEnrich19
	90	215	<50	123	221	124	FJ808727	*Aeromonas veronii strain QXL0711B* 16S ribosomal RNA gene	99%	
*Shewanella*										
FL2B*	496	574	<50	127	49	123	AF387349	*Shewanella* sp.*184* 16S ribosomal RNA gene, partial sequence	99%	AP-FeEnrich20
*Enterobacteriaceae*										
GG3A*	493	373	206	127	49	123	AY394724	*Serratia plymuthica RVH1* 16S ribosomal RNA gene, complete sequence	98%	AP-FeEnrich21
FG4B, GG1A, GG3B, GM2B, HL3A*	494	371	<50	127	49	123	HQ407251	*Serratia fonticola strain G75* 16S ribosomal RNA gene, partial sequence	98%	AP-FeEnrich22
HG3B*	494	371	<50	127	49	123	DQ068814.1	Uncultured bacterium clone f6h4 16S ribosomal RNA gene, partial sequence	99%	AP-FeEnrich23
							AB353048	*Klebsiella oxytoca* gene for 16S ribosomal RNA, partial sequence, strain: No.8	99%	
GM3A,										
**SPIROCHETE**										
FA1B, FA2B, FA4A*, FA4B, FL1B, FL2A*, FL2B, FL3A*, FL4A, FG1A*, FG2A*, FG2B, FG3A*, FG3B, FG4B, FM1A, FM1B, FM2A, FM2B, FM3A, FM4B, GL3A*, GL3B, GG1A, GG1B, GG2A*, GG3A*, GG3B, GM1A, GM1B, GM2A, GM2B, HG3A, HL3A*	210	62	210	126	146	125	GU080088 AY695839	Bacterium enrichment culture clone N47 isolate 2 16S ribosomal RNA gene *Spirochaetes bacterium SA-8* 16S ribosomal RNA gene, partial sequence	99%99%	AP-FeEnrich24

*Major peaks in the T-RFLP profiles were assigned to phylogenic groups by conducting *in silico* digestions of clone sequences. The columns MspI, *Hha*I, and *Hae*III represent the length of *in silico* T-RF sites for clones*.

Samples with T-RF sites matching a cloned sequence (*) were placed under the respective bacteria with sample nomenclature as: First letter = Fe(III) oxide, Second Letter = Carbon Source, and Number = Dilution. For example, in the first row under *Geothrix* FL3 stands for ferrihydrite with lactate in the 3rd dilution. Because clones most closely related to *Aeromonas* spp. and *Serratia fonticola/Klebsiella oxytoca* have the same restriction site for the six T-RFs obtained in this study it was no plausible to assign the those sample peaks to either group.

### Statistical analysis of TRFLP patterns in Fe(III)-reducing enrichments

**Figure 2:**
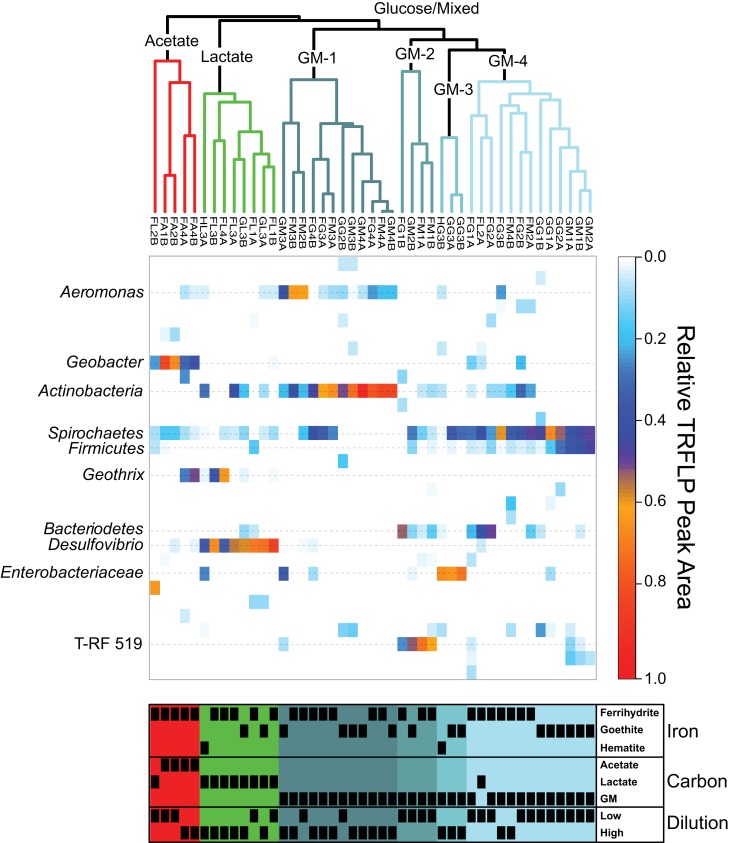
**Top: average agglomerative (UPGMA) clustering based on Bray–Curtis dissimilarity of *Msp*I relative abundance T-RFLP profiles**. Colors of the clusters indicate groupings based on cutting the dendrogram at BC_dis_ = 0.7. GM is abbreviated for combined glucose and mixed carbon source. Middle: heatmap depicting the relative abundance of T-RF peak area for each enrichment (column) with identified phylogenetic affiliations (rows) labeled on the left. Bottom: annotation of enrichments indicating the presence (black box) or absence (blank) of enriched communities within each cluster as a function of Fe oxide, carbon source, and dilution. The dilution variable is presented as either low (first or second) or high (third and fourth) sediment dilution.

The community composition of the 45 enrichments was analyzed using the Bray–Curtis dissimilarity index (BC_dis_). Hierarchical clustering analysis was relatively unbiased at reproducing the pairwise Bray–Curtis dissimilarities (cophenetic correlations = 0.82) and revealed a high degree of dissimilarity among all enrichments, with the largest cophenetic dissimilarity (CD) = 0.9059 (Figure 2). Further examination of the dendrogram by clustering into three distinct groups revealed that enrichments grouped based on carbon source utilization. Non-parametric ANOVA (Kruskal–Wallis rank sum test) confirmed that these groupings were indeed different with respect to carbon source (*p* < 0.05).

The first cluster (CD = 0.6996; red grouping), with five enrichments, contained all four acetate enrichments and an additional lactate enrichment (Figure 2). Within this group, the four acetate samples were more similar to each other (CD = 0.5995) than to the single lactate sample. These enrichments contained between 4 and 7 T-RFs with two peaks being present in all samples, corresponding to the phylum *Spirochaetes* and genus *Geobacter*. While *Spirochaetes* were present in all samples, their relative peak area (4–15%) was far lower than *Geobacter* (23–80%). Within the acetate samples in this cluster, the relative peak area corresponding to *Geobacter* sp. were greatest (66–80%) in the low dilution (first and second) samples and decreased (30–37%) in higher dilution (fourth). The decrease in the relative abundance of *Geobacter* peaks in these higher dilution enrichments corresponded to a concurrent increase in peak area corresponding to the genus *Geothrix* within the phylum *Acidobacteria* (26–49%). A diversity of *Geobacter* species are well-known for the ability to couple the oxidization of acetate to the reduction of ferrihydrite and therefore this organism was likely responsible for the reduction of Fe(III) in these enrichments. Interestingly, this cluster contained only ferrihydrite and not the more recalcitrant Fe(III) oxides goethite or hematite. In fact, within the 45 reduced enrichments there was a significant difference (*p* < 0.05) in *Geobacter* sp. abundance based on carbon source and Fe(III) oxide type, with T-RFs from this organism dominating in enrichments containing ferrihydrite and acetate.

The second cluster (CD = 0.5480; green grouping) contained eight enrichments, all of which contained lactate as the carbon source (Figure 2). Within this cluster there were five ferrihydrite enrichments, however, unlike the acetate dominated cluster, enrichment cultures capable of reducing the more recalcitrant Fe(III) phases goethite (two enrichments) and hematite (one enrichment) were also present. Enrichments within this cluster contained between 2 and 8 T-RFs with three phylogenetic groups present in five or more samples (*Geothrix*, *Spirochaetes*, *Desulfovibrio*). Six enrichments contained *Spirochaetes* sp. where they made up 4–11% of the relative peak area. *Geothrix* sp. were present in five enrichments and made up 1–63% of the relative area. Similar to the acetate cluster, the relative abundance of *Geothrix* sp. was higher (35 and 63%) within higher dilution (third and fourth) enrichments. Of the three phylogenetic groups present in most enrichments, only *Desulfovibrio* was present in all eight enrichments, including all three Fe(III) oxides, and represented the largest relative peak area (32–82%). Non-parametric ANOVA confirmed that carbon source (*p* < 0.05) but not Fe oxide type was significant in selecting for *Desulfovibrio* species within the enrichments.

The last cluster was the largest and most diverse with 32 enrichments containing all 16 mixed carbon and 15 glucose enrichments with one additional lactate enrichment (Figure 2; blue-gray groupings). However, the largest cophenetic dissimilarity for this cluster was high (CD = 0.8471) and was therefore divided further into four distinct groups with BC_dis_ < 0.7. Analysis of these four clusters showed that they correspond to samples enriched in a diverse set of organisms including *Aeromonas*, *Actinobacteria*, *Bacteroidetes*, *Firmicutes*, *Spirochaetes*, *Enterobacteriaceae*, and an unknown peak at 519 bp. The first grouping (CD = 0.6130; GM-1) contained 12 enrichment cultures, seven of which were enriched on ferrihydrite and five on goethite. While Fe(III) phase did not seem to play a role in selecting for communities in this cluster, higher sediment dilutions were clearly selected for – two enrichments from the second dilution, five from the third dilution, and five from the fourth dilution. The 12 enrichments in this group contained 2–7 T-RFs with two organisms, *Aeromonas* (0–62% peak area), and *Actinobacteria* (19–94% peak area) present in 11 and 12 samples, respectively. In the second group (CD = 0.6513; GM-2), four enrichment cultures contained 5–8 T-RFs and all four were enriched in an unknown T-RF at 519 bp (25–71% relative peak area) and *Bacteroidetes* (5–51% relative peak area). The unknown peak at 519 bp closely matched (five of six T-RF peaks) a *Clostridium* sp. sequence from another enrichment, however, it was left unknown since direct phylotype accounting for all six peaks was not sequenced. Interestingly, the third group in the GM cluster contained three enrichments on the recalcitrant Fe(III) oxides (two goethite and one hematite) that clustered closely together (CD = 0.3440; GM-3) and showed substantial amounts of reduction (56–67%; Figure 1). Only two organisms were present in all three enrichments, *Spirochaetes* and *Enterobacteriaceae*, with members of the *Spirochaetes* representing 2–35% of the relative peak area, while members of the *Enterobacteriaceae* family represented 63–69%. Indeed, non-parametric ANOVA confirmed that Fe(III) oxide phase played a role in selecting for *Enterobacteriaceae* (*p* < 0.05). Only one of the eight enrichment cultures hosting *Enterobacteriaceae* contained ferrihydrite as the Fe(III) source and *Enterobacteriaceae* sp. were in relatively low abundance. Finally, the last cluster (CD = 0.6038) contained 13 enrichments mostly from low dilution samples (five first dilution, six second dilution, one third dilution, one fourth dilution). Two T-RFs corresponding to *Firmicutes* (1–46% relative peak area) and *Spirochaetes* (29–67% relative peak area) were present in all 13 enrichments, while *Actinobacteria* (5–30%) and *Bacteroidetes* (4–49%) were both present within six enrichment cultures. Within this cluster, Fe mineralogy also seemed to play a role in selecting for community structure as the six goethite samples were more similar to each other than they were to the other seven ferrihydrite samples.

### Bacterial community composition as a function of Fe oxide, carbon source, and dilution

**Figure 3:**
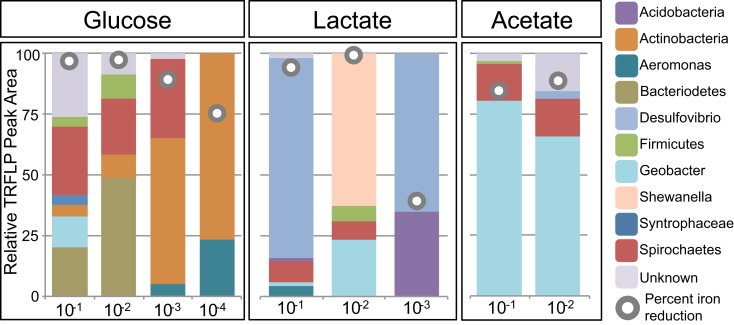
**Relative peak area (%) of *Msp*I T-RFs for ferrihydrite enrichments as a function of dilution and carbon source**. T-RF peak area was used to obtain relative percentages of each phylogenetic group. The gray circles indicate the percent Fe(III) reduced in the enrichment.

**Figure 4:**
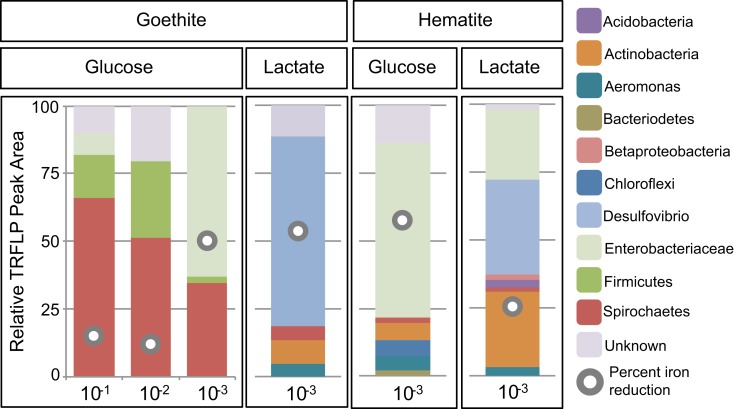
**Relative peak area (%) of *Msp*I T-RFs for goethite and hematite as a function of dilution and carbon source (glucose or lactate)**. T-RF peak area we as used to obtain relative percentages of each phylogenetic group. The gray circles indicate the percent Fe(III) reduced in the enrichment.

Select enrichments from the dendrogram (Figure 2) are shown in more detail (Figures 3 and 4), in order to more clearly represent the community composition dynamics as a function of carbon source, dilution, and Fe oxide. When glucose was provided as the carbon source for ferrihydrite enrichments, bacterial populations consisted of dominantly three members that varied with sediment dilution (Figure 3). The enriched bacterial population shifted from dominantly *Bacteroidetes* (29–40%) and *Spirochaetes* (39–28%) at low dilutions to dominantly *Actinobacteria* (77%) and *Aeromonas* (23%) at higher dilutions (Figure 3). This species transition with dilution was seen in the dendrogram as a shift from the low dilution containing GM-2 and GM-4 groups to the GM-1 group containing higher dilution enrichments (Figure 2). This shift in population corresponded to a moderate decrease (∼25% decrease) in observed ferrihydrite reduction (Figure 1).

Ferrihydrite-reducing communities enriched on lactate had a greater variability in community composition, evidenced by the fact that they were observed in all three major clusters in the dendrogram (Figure 2). In comparing the first and second dilution where Fe(III) reduction is nearly complete, the communities shifted from dominantly sulfate-reducing *Desulfovibrio* sp. (76%) to the DIRM *Shewanella* (56%) and *Geobacter* spp. (26%; Figure 3). In the third dilution enrichment culture, where Fe(III) reduction was substantially lower (39%), *Desulfovibrio* sp. dominated again but were accompanied by *Geothrix* sp. (43%).

When acetate was provided as the carbon source, *Geobacter* sp. dominated the reduced ferrihydrite enrichments (Figure 3) and a substantial amount of Fe(III) reduction (84–88%) was observed.

Within goethite enrichments containing glucose as the carbon source, peaks corresponding to *Spirochaetes* (35–66%), *Firmicutes* (3–28%), and *Enterobacteriaceae* (0–63%) were observed (Figure 4). *Spirochaetes* (51–66%) and *Firmicutes* (16–28%) dominated the first and second dilution enrichments cultures, where 18–21% Fe(III) reduction was observed. In the 10^−3^ dilution enrichment, however, where more substantial Fe(III) reduction (56%) was observed (Figure 1), *Enterobacteriaceae* was present (63%) alongside *Spirochaetes* (35%). This change in community structure was also seen in the dendrogram as these enrichments move from the low dilution GM-4 group to the GM-3 grouping containing enrichments relatively high in *Enterobacteriaceae* (Figure 2). For lactate enrichments supporting goethite reduction (10^−3^ dilution), the bacterial community was composed of *Desulfovibrio* (70%), *Actinobacteria* (9%), *Aeromonas* (5%), and *Spirochaetes* (5%).

For hematite, substantial Fe(III) reduction was only observed in the third sediment dilution enrichment culture containing either lactate or glucose as the carbon source (Figure 1). Within the glucose enrichment, six organisms were identified with *Enterobacteriaceae* sp. representing the majority (64%) of the community (Figure 4). When lactate was provided as the carbon source, the community within the third dilution enrichment culture was dominated by *Desulfovibrio* (35%)*, Actinobacteria* (28%), and *Enterobacteriaceae* (26%).

### Distribution and phylogenetic identity of primary species within enrichment cultures

**Figure 5:**
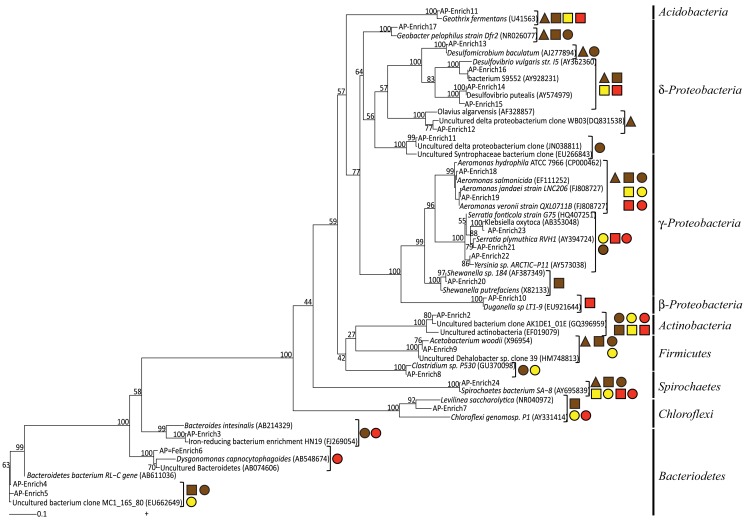
**Maximum likelihood 16S rRNA phylogenetic tree for Fe(III)-reducing enrichment clones**. Shapes indicate carbon sources (▲ = acetate ■ = lactate ● = glucose) and color indicates Fe(III) oxide type (red = hematite, yellow = goethite, brown = ferrihydrite) for which those microorganisms were enriched on. Bootstrap values (*n* = 1000), as percentages, are indicated at nodes.

While some microbial groups appeared to proliferate within these enrichments indiscriminately, others appeared to be enriched under select conditions (C, Fe oxide, dilution). For instance, the common Fe(III)-reducer *Geobacter* sp. was obtained within Fe(III)-reducing enrichments containing primarily acetate and only ferrihydrite. The enriched species shared 97% sequence identity to *Geobacter pelophilus strain Dfr2* (Straub and Buchholz-Cleven, 2001; Figure 5). In contrast, bacteria within the *Enterobacteriaceae* family were predominantly enriched on the more crystalline Fe(III) phases goethite and hematite. Species within the genus *Serratia* were enriched on both goethite and hematite, with 98% sequence identity to *Serratia plymuthica* RVH1 and to *Serratia fonticola* G75. Enriched *Enterobacteriaceae* on hematite supplemented with glucose belonged to a different genus and had 98% sequence identity to *Klebsiella oxytoca*. Thus, in these cases, both carbon and Fe oxide type selected for the putative Fe(III)-reducing organisms within the enrichment cultures.

In some cases, carbon source played a much larger role in selecting for the Fe(III)-reducing community rather than the Fe oxide phase. For instance, the relative abundance of *Desulfovibrio* species were shown to be statistically different based on carbon source, with the majority being enriched on lactate. The dominant *Desulfovibrio* species in these enrichments were similar (99% sequence identity) to the known sulfate-reducing bacterium, *D. putealis* strain B7-43 (Basso et al., 2005; Figure 5). *D. putealis* was enriched on all three Fe(III) oxides, being the only *Desulfovibrio* species enriched on hematite and the closest cultured representative for 2 T-RF enriched on ferrihydrite (the percent sequence identity varied from 96–99%; Table 1). However, in two enrichments amended with lactate, one ferrihydrite and one goethite, the majority (61 and 48%) of the T-RF peak area was most similar to *D. vulgaris* (95% sequence identity) with the remainder of the *Desulfovibrio* relative peak area belonging to *D. putealis*.

Some bacterial groups showed a cosmopolitan distribution, seeming to enrich regardless of carbon and Fe oxide provided. Further, these groups in some cases showed little to no species-level diversity as a function of Fe(III) oxide or carbon. For instance, despite *Spirochaetes* being present in 34 of the enrichments (76%), including various carbon and Fe(III) oxide substrates, all sequences were 99% similar to the *Spirochaetes* isolate SA-8 (Bonin, 2005; Figure 5). Likewise, *Actinobacteria* sp. were in 25 enrichments (56%) including all three Fe(III) oxides and two carbon sources (glucose, lactate), yet all species were closely related (∼99%) to each other and had no cultured representative with greater than 90% sequence identity (*Olsenella* sp. F0004; 88% similarity). Similarly, seven enrichments with acetate and lactate as carbon source but varying in Fe(III) oxide contained members of the *Acidobacteria* phylum with all species having 97% sequence identity to *Geothrix fermentans* strain H5 (Coates et al., 1999). Lastly, bacteria within the *Aeromonas* genus were present in 18 enrichments including all carbon sources and Fe(III) oxides. The enriched *Aeromonas* bacteria are represented by a wide range of species with ∼99% similarity (Figure 5 and Table 1), with no clear species-level preference for Fe oxide or carbon source.

## Discussion

Within soil and sedimentary environments, complex organic matter breaks down into numerous metabolic byproducts fueling fermentation and respiration that may be coupled either directly or indirectly to Fe(III) reduction (Lovley et al., 2006). Despite the obvious significance of microbial activity in the reduction of Fe(III) in non-sulfidogenic environments, the role that carbon source and Fe(III) oxide mineralogy play in controlling community structure and ultimately the reduction of varying Fe(III) phases is still poorly understood. This study represents the first large-scale cultivation effort to identify organisms (and/or communities) responsible for Fe(III) reduction as a function of carbon, Fe(III) oxide structure (hence bioavailability), and sediment dilution. We observed statistically supported clustering of enriched Fe(III)-reducing communities primarily based on the carbon source provided. These findings are consistent with biostimulation experiments in which the choice of electron donor supported diverse and distinct communities capable of metal reduction (Akob et al., 2008; Burkhardt et al., 2009). Secondary to carbon, however, both sediment dilution and Fe(III) oxide shaped Fe(III)-reducing communities and dictated putative Fe(III) reduction pathways (e.g., via sulfate reduction) observed here. In fact, differences in community structure as a function of carbon source and sediment dilution dictated not only the amount of Fe(III) reduced but also whether more recalcitrant Fe phases were available for reduction. Similarly, the crystallinity of Fe oxides has also been shown to affect which organisms assimilate ^13^C-labeled acetate (Hori et al., 2010).

Acetate is considered to be a key product of the breakdown of complex organic matter and one of the primary carbon substrates fueling Fe(III) reduction in reduced soils and sediments (Lovley and Phillips, 1989). Here, when acetate was provided as the electron donor within sediment enrichments, only the reduction of the poorly crystalline Fe oxyhydroxide ferrihydrite was observed. In these enrichments the well-known DIRM *Geobacter* proliferated and a substantial amount (up to 89%) of Fe(III) reduction occurred (Figures 1–3). Organisms within the *Geobacter* clade are dominant metal-reducing organisms within many sediments (Lovley et al., 2004). While *Geobacter* species have the demonstrated ability to reduce various Fe(III) sources, including ferrihydrite, their ability to reduce more crystalline phases is limited (Lovley and Phillips, 1988). Indeed, although *Geobacter* sp. are present in the sediments here (based on its occurrence in ferrihydrite enrichments), little to no Fe(III) reduction (0–6%) of goethite or hematite was coupled to the oxidation of acetate (Figure 1). These findings are in line with previous enrichments of rice paddy soils containing ^13^C-labeled acetate that showed *Geobacter* sp. assimilated ^13^C-labeled acetate in both ferrihydrite and goethite enrichments, yet Fe(III) reduction was only measured for ferrihydrite (Hori et al., 2010). Thermodynamic calculations indicate that reduction of goethite coupled to acetate oxidation is, in fact, unfavorable (Δ*G*′ = ∼0 kJ) under our enrichment conditions (pH 7.3; [acetate] = 10 mM; [Fe^2+^] = 5 μM; [${\text{HCO}}_{3}^{-}$] = 10 mM). Thus, in mature sediments where more stable and hence recalcitrant Fe(III) oxides dominate, acetate oxidation will likely not support substantial Fe(III) reduction.

*Geobacter* species may also be involved in ferrihydrite reduction when lactate is provided as the electron donor. In the 10^−2^ dilution enrichment containing lactate and ferrihydrite, *Geobacter* and *Shewanella* spp. accounted for greater than 75% of the population (Figure 2). *Shewanella* species have a wide metabolic plasticity, including the dissimilatory reduction of various Fe(III) substrates (Myers and Nealson, 1988). Since *Shewanella* species incompletely oxidize lactate to acetate, the presence of both *Shewanella* and *Geobacter* species may, in fact, suggest a syntrophy between the two organisms under these conditions. Under most lactate-oxidizing conditions, however, Fe(III) reduction is not linked to known dissimilatory metal-reducing organisms and instead appears to be linked to sulfate-reducing and fermenting organisms.

**Figure A1:**
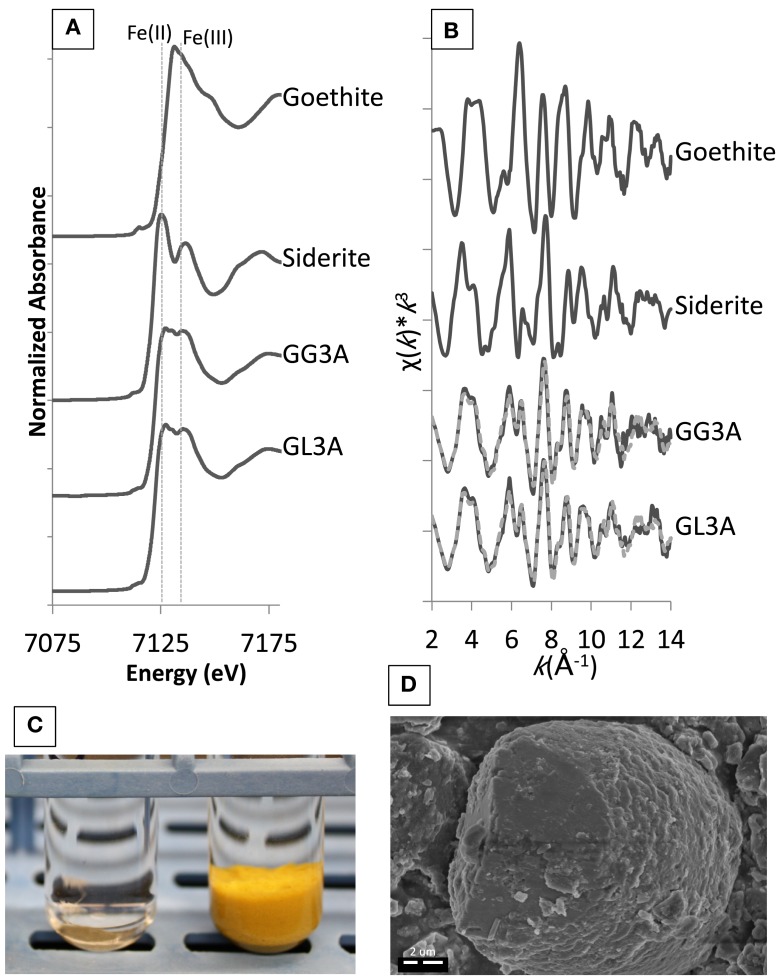
**The top images show (A) XANES and (B) EXAFS spectra for secondary minerals formed within goethite enrichments in the presence of glucose or lactate**. The reference spectra goethite and siderite are also included to illustrate the relative binding energies of Fe(II) and Fe(III) and line shape for the two mineral phases. The bottom images illustrate **(C)** the visual disappearance of the goethite mineral after incubation and **(D)** observation of secondary siderite grains via SEM.

In fact, Fe(III) reduction appears to be correlated to the presence of members of the genus *Desulfovibrio* (Figures 2–4) when lactate is the carbon source. *Desulfovibrio* species are abundant, widespread organisms within the δ*-proteobacteria* known for their ability to couple the oxidation of lactate to the reduction of sulfate to sulfide. The genus, however, is diverse in its ability to use a variety of other electron donors (e.g., lactate, acetate, pyruvate) and acceptors [e.g., Fe(III), ${\text{NO}}_{3}^{-}$; Bale et al., 1997]. Despite sulfate concentrations being similar to the modest levels typical of freshwater environments (200 μM), *Desulfovibrio* spp. were found in 90% of the Fe(III)-reducing enrichments containing lactate, with two species dominating (*D. putealis* and *D. vulgaris*). Fe(III) reduction within these enrichments was likely a consequence, at least in part, to sulfide formation and subsequent reaction between sulfide and Fe(III). In fact, within these enrichment cultures, sulfate was not detected indicating that sulfate was completely consumed. It has been previously demonstrated that Fe(III) reduction can be sustained on catalytic amounts of sulfur (Straub and Schink, 2004). Given the low S to Fe ratio here (1:125), Fe-S precipitation could be impeded by scavenging of Fe(II) from solution thus allowing sulfur to remain in the aqueous phases and available for continued Fe-S cycling. Indeed, extensive siderite (FeCO_3_) precipitation was observed within the goethite enrichments (Figure A1 in Appendix). Thus, for a catalytic S cycle to be responsible for the amount of Fe(III) reduction observed here, sulfide would need to: (1) be abiotically re-oxidized by Fe to a S intermediate used by an organism in the consortium, and not elemental sulfur as previously suggested (Pyzik and Sommer, 1981; Poulton et al., 2004) or (2) if oxidized to elemental sulfur, a second organism capable of reducing elemental sulfur or taking it to a higher oxidation state (i.e., through disproportionation) would need to be present in the enrichment (Thamdrup et al., 1993).

Alternatively, given that there are at least two known *Desulfovibrio* species that can use Fe(III) as an electron acceptor (Lovley et al., 1993; Bale et al., 1997; Park et al., 2008), direct enzymatic Fe(III) reduction within these enrichments could be operative. Yet, based on thermodynamic calculations specific to conditions within these enrichments (pH 7.3; [lactate] = 10 mM; [acetate] = 1 μM; [Fe^2+^] = 5 μM; [${\text{HCO}}_{3}^{-}$] = 10 mM; [${\text{SO}}_{4}^{2-}$] = 200 μM; [HS-] = 1 μM), the reduction of ${\text{SO}}_{4}^{2-}$ coupled to incomplete oxidation of lactate is more favorable (Δ*G*′ = ∼101 kJ) than goethite (Δ*G*′ = ∼86 kJ) even with the modest (200 μM) levels of ${\text{SO}}_{4}^{2-}$ provided. Indeed, these observations are in line with previous calculations that used a partial equilibrium approach to predict a preference for sulfate reduction within environments dominated by more crystalline (e.g., hematite) Fe oxides (Postma and Jakobsen, 1996) and explain similar field observations in which sulfate reduction happened before or simultaneously with Fe(III) reduction (Jakobsen and Postma, 1999). Further, by profiling mRNA transcripts of putative Fe(III)- and key sulfate-reducing genes, recent research suggested that SRBs play a role in suppressing *Geobacter* sp. activity through competition for acetate even during active Fe(III)-reducing conditions (Akob et al., 2012).

As might be expected, the glucose and the mixed carbon enrichments showed the greatest richness (number of T-RFs) and diversity, as evidenced by the many sub-clusters in the dendrogram (Figure 2) as well as the large cophenetic dissimilarity. Interestingly, while the mixed carbon source contained all three carbon compounds (glucose, lactate, and acetate), mixed carbon enrichment cultures always clustered closely with the glucose enrichments suggesting that even in the presence of lactate and acetate, the most energetic carbon source, glucose, selected for the dominant community structure. Glucose and mixed carbon enrichment cultures were dominated by known fermenting organisms, including *Firmicutes* and *Enterobacteriaceae*. Despite the focus on dissimilatory Fe(III) reduction within the last few decades, some studies have agreed with earlier research implicating fermentative bacteria as relevant organisms in environmental Fe(III) reduction (see for example, Jones et al., 1984; Petrie et al., 2003; Hansel et al., 2008). It remains unclear if Fe(III) simply provides a sink for excess electrons or is used for energy conservation (Dobbin et al., 1999) by fermenting organisms. Yet, recent evidence supports earlier claims that Gram-positive fermentative organisms are capable of indirect and direct enzymatic electron transfer to Fe(III) oxide surfaces as well as solid-phase electrodes (Wrighton et al., 2011a,b).

The more complex carbon sources also supported Fe(III) reduction along a wide dilution gradient. The enriched Fe(III)-reducing population varied with sediment dilution, likely reflecting specific environmental niches for the putative Fe(III)-reducing communities. For instance, ferrihydrite enrichment cultures supplemented with glucose showed substantial reduction up to a 10^−4^ sediment dilution (Figure 1). At higher dilutions (GM-1 cluster), *Actinobacteria* sp. were the dominant organisms and may have been responsible for ferrihydrite reduction, yet these phylotypes were present in low abundance at lower dilutions (Figure 2). This distribution suggests that *Actinobacteria* species were in high abundance within the natural sediment, yet were slow(er) growing under these conditions and therefore outcompeted for resources by faster growing organisms in low dilutions. *Actinobacteria* sp. are common in sediments where they demonstrate diverse metabolisms ranging from propionic acid fermentation to acetogenesis (Madigan et al., 2002). While metal-reducing capacity by *Actinobacteria* has not been confirmed, they are frequently detected within active metal-reducing communities (Akob et al., 2007; Lin et al., 2007; Wang et al., 2009) and have also assimilated ^13^C-labeled ethanol during the metal-reducing phase of sediment incubations (Akob et al., 2011).

Interestingly, members of the *Enterobacteriaceae* family also showed a strong dependence on sediment dilution and were observed primarily (88%) on goethite or hematite enrichments. Members of this family are phylogentically diverse with organisms in the *Serratia* and *Klebsiella* clades being known for mixed acid/butanediol fermentation (Madigan et al., 2002). In fact, fermentation products, including acetate, lactate, succinate, propionate, and formate, were abundant within these enrichments (Table A1 in Appendix). While the current data cannot directly connect these organisms to Fe(III) reduction observed here, given the low diversity in these enrichments and the correlation between the presence of these species with increased Fe(III) reduction (Figure 4), they likely play a role. Indeed, members of *Serratia* have previously been implicated in the reduction of hematite (Ottow, 1970). The observation of goethite and hematite reduction only at one specific dilution (10^−3^) within these enrichments (Figure 1), suggests that these organisms occupy specific environmental niches.

While the dominant putative Fe(III)-reducing organisms in the enrichment cultures appeared to be *Geobacter* sp., *Desulfovibrio* sp., and a number of fermenting organisms (depending on dilution and Fe oxide), various other organisms were present whose role in Fe(III) reduction remains unknown. For instance, while *Geothrix*, *Aeromonas*, and *Spirochaetes* spp. have demonstrated abilities to reduce Fe(III), their presence does not seem to correlate with Fe(III) reduction capacity (Figures 3–4) putting into question their role in Fe(III) reduction under these conditions. *Geothrix* sp. are present in seven enrichments, six of which are higher dilutions (10^−3^ to 10^−4^). Their relative peak area decreases with increasing crystallinity, comprising only a maximum of 3% on hematite and goethite but up to 63% on ferrihydrite. *Geothrix* species have been shown to couple oxidation of both lactate and acetate to the reduction of poorly crystalline Fe oxides (Coates et al., 1999), where Fe(III) chelation is possibly involved (Nevin and Lovley, 2002). Members of the genus *Aeromonas* and *Spirochaetes* were observed within all Fe oxide and carbon conditions. *Spirochaetes* species have a demonstrated ability to reduce Fe(III) (Vu et al., 2004) and the species enriched here is similar to an isolate (SA-8) obtained from an Fe(III)-reducing enrichment (Bonin, 2005). *Spirochaetes* sp. also have the ability to ferment sugars via the glycolytic pathway, convert H_2_ and CO_2_ to acetate (acetogens), and fix nitrogen (Leadbetter et al., 1999; Madigan et al., 2002; Vu et al., 2004). Similarly, *Aeromonas* sp. have demonstrated the ability to couple growth to Fe(III) reduction (Knight and Blakemore, 1998; Scala et al., 2006). Yet, our findings support previous research suggesting that the direct reduction of solid-phase Fe(III) by these organisms is minimal (Knight et al., 1996; Knight and Blakemore, 1998). Instead, *Aeromonas* have the capacity to grow facultatively by either fermentation or anaerobic respiration with other electrons acceptors (e.g., fumarate; Knight and Blakemore, 1998).

While these organisms (*Aeromonas* and *Spirochaetes*) were present in high proportions and their direct involvement in Fe(III) reduction is unknown, they may play a supporting role in the Fe(III)-reducing community. For instance, *Aeromonas veronii* stimulated Fe(III) reduction in co-cultures with *Shewanella alga* through the dissimilation of citrate to formate, which *S. alga* used as an electron donor (Knight et al., 1996). Furthermore, a *Spirochaetes* species similar (98% sequence identity) to the enriched species in this paper was a stable member of a hydrocarbon-degrading consortium, where it was predicted that it served a syntrophic role in supporting sulfur cycling (Selesi et al., 2010). While unknown at this time, this type of synergistic interaction may explain the high abundance and widespread distribution of *Aeromonas* and *Spirochaetes* species within the enrichment cultures.

Our findings support previous research concluding that dissimilatory metal-reducing organisms are responsible for the reduction of labile Fe(III) phases, primarily in the presence of acetate. We have shown here however that enrichment cultures supporting substantial goethite or hematite reduction coincided with increased relative abundances of *Firmicutes*, *Enterobacteriaceae*, and *Desulfovibrio* spp. (Figures 2–4). These findings suggest that the reduction of more crystalline Fe oxides within natural environments, as observed previously (e.g., Stucki et al., 2007), is likely a consequence of fermentation and/or sulfate reduction and not dissimilatory metal reduction. Interestingly, carbon source utilization and resource competition may ultimately dictate active Fe(III)-reducing microbial populations and operative Fe(III) reduction pathways, with increased metal reduction in areas containing more complex (higher) carbon sources (e.g., glucose). In young, dynamic environments where ferrihydrite is likely to exist, the growth and activity of DIRMs would be expected. As ripening and/or dissolution-reprecipitation leads to more stable Fe(III) (hydr)oxide end members, however, we expect a shift to sulfate-reducing and fermenting microbial communities which may be capable of directly or indirectly reducing these more stable Fe(III) phases. In fact, minimal Fe(III) reduction may be possible in aged mature soils and sediments that are dominated by lower carbon sources and stable Fe(III) oxides. Ultimately, these findings have implications for our understanding of the cycling of Fe and degradation of carbon in dynamic Fe(III)-reducing environments.

## Conflict of Interest Statement

The authors declare that the research was conducted in the absence of any commercial or financial relationships that could be construed as a potential conflict of interest

## Footnotes

^1^http://www.R-project.org/, 2012

## Appendix

### Secondary mineralization

#### Methods

Secondary Fe phases formed following the reduction of Fe(III) were investigated using X-ray absorption spectroscopy [both X-ray absorption near edge spectroscopy (XANES) and extended X-ray absorption fine structure (EXAFS) spectroscopy] and scanning electron microscopy (SEM). Before analysis, samples were dried anaerobically in a glove bag. For XAS, the samples were then mounted on a Teflon holder and sealed with Kapton polyimide film. Following data collection at beam line 11–2 (Stanford Synchrotron Radiation Lightsource) a set of Fe reference standards were fit to the data (*k^3^* weighted linear combination fitting; k-range 2–14Å) using the program SIXPack (Webb, 2005; For further details see Hansel et al., 2003).

For SEM, samples were mounted on SEM stubs with double-sided carbon tap and sputter-coasted with Au prior to imaging. SEM was performed at the Harvard University Center for Nanoscale Systems using a Zeiss FESEM Supra 55VP with a high efficiency in-lens secondary electron detector and equipped with an EDAX Genesis (EDS) system.

#### Results

The reduction of ferrihydrite and goethite leads to the precipitation of the secondary Fe phase, siderite (Fe^II^CO3) regardless of the carbon source or dilution. For instance, for the goethite enrichments amended with glucose or lactate where substantial Fe(III) reduction is observed (10^−3^ dilution), there is a shift in the XANES spectra to lower energies indicating a higher proportion of Fe(II) to Fe(III) within the solid-phase products (Figure A1A). The EXAFS spectra are best fit using a linear combination comprised of 61% siderite and 39% goethite for the glucose enrichments and 62% siderite and 33% goethite for the lactate enrichments (Figure A1B). These percentages are in good agreement with the percent Fe(II) found via ferrozine (see Figure 1).

In addition to spectroscopic evidence, visible indications of reduction were also noted as demonstrated by comparing the solid-phase appearance before (Figure A1C – right) and after (Figure A1C – left) incubation. This striking difference was seen over continuous transfers for goethite in the third and fourth dilution of lactate and glucose, providing further confirmation of repeated reduction of goethite in these enrichments. Microscopic analysis reveals that the siderite particles are small (average 10 μm diameter) and have a botryoidal morphology similar to frequently observed biogenic siderite (Figure A1D). Indeed, EDS indicated that these particles contained only iron and carbon.

### Organic carbon analysis

#### Methods

Selected organic acids (acetate, butyrate, citrate, formate, fumarate, gluconate, lactate, malate, maleate, malonate, oxalate, propionate, pyruvate, succinate, tartrate) and major ions (${\text{SO}}_{4}^{-2}$, Cl) were analyzed via suppressed anion chromatography with conductivity detection using a Dionex ICS-2000 (AS11 Column) with a KOH eluent generator. An eluent gradient method was employed (flow rate 1.5 mL min^−1^): beginning for 6 min at 1 mM, followed by a linear ramp to 30 mM over 8 min, another linear ramp to 60 mM over 4 min, followed by 5 min re-equilibration at 1 mM between samples. Samples were diluted 1:10 in DI water and frozen until analysis. Standard solutions were made gravimetrically and a full size range of standards (1000, 250, 100, 50, and 10 μM) was analyzed approximately every 20 samples (r^2^ typically >0.999) while a single 100 μM standard was analyzed every 10 samples to account for any potential instrument drift. Based on repeated analysis of standard solutions, standard errors for the compounds detected and quantified in our enrichments, lactate, acetate, succinate, propionate, butyrate, formate, and ${\text{SO}}_{4}^{-2}$ were ±20, 206, 414, 367, 81, 27, and 29 μM, respectively (Table A1).

**Table A1:** **Organic acids produced in glucose and lactate enrichments**.

Mineral	Dilution	Lactate mM (±0.02 mM)	Acetate mM (±0.2 mM)	Succinate mM (±0.4 mM)	Propionate mM (±0.4 mM)	Butryate mM (±0.08 mM)	Formate mM (±0.03 mM)
**GLUCOSE AMENDED**							
Ferrihydrite	10^−1^	–	–	–	0.9	–	–
Ferrihydrite	10^−2^	***	14.2	***	2.6	2.0	***
Ferrihydrite	10^−3^	–	9.2	***	8.4	***	–
Ferrihydrite	10^−4^	0.5	9.8	1.8	***	–	2.8
Goethite	10^−1^	–	19.3	–	5.1	***	***
Goethite	10^−2^	***	13.5	–	4.4	***	***
Goethite	10^−3^	***	24.7	1.2	0.4	***	***
Hematite	10^−3^	–	3.3	***	***	–	***
**LACATE AMENDED**							
Ferrihydrite	10^−1^	6.0	3.9	***	***	***	0.3
Ferrihydrite	10^−2^	–	13.5	–	***	***	***
Ferrihydrite	10^−3^	2.6	7.3	***	***	–	***
Goethite	10^−3^	7.8	1.3	–	***	–	***
Hematite	10^−3^	3.5	4.9	***	0.5	***	0.3

*–, Not detected; ***, trace amount detected, not quantifiable*.

*Error estimates are calculated as standard error multiplied by factor for analysis of 10× diluted samples*.

#### Results

All enrichment cultures containing lactate and glucose exhibited varying accumulation of acetate, lactate, succinate, propionate, butyrate, and formate, reflecting underlying changes in community metabolism (Table A1). Enrichments grown on lactate showed variability in both the amount of lactate consumed and the amount of acetate accumulated as a product of incomplete lactate oxidation. While some amount of lactate consumption was observed in all enrichments shown in Table A1, initial levels of lactate were drawn down completely in only one enrichment, ferrihydrite (10^−2^). Further, low levels of succinate, propionate, butyrate, and formate were observed (though not quantifiable) across all lactate enrichments, with accumulation of up to 0.3 mM formate in ferrihydrite (10^−1^) and hematite (10^−3^) and 0.5 mM propionate in hematite (10^−3^).

Enrichments on glucose also demonstrated considerable variability in production and accumulation of organic acids. In general more acetate was produced in the goethite enrichments (9.7–24.7 mM) followed by ferrihydrite (0–14.2 mM) and then hematite (3.3 mM). No acetate formation was observed in the ferrihydrite 10^−1^ dilution. Variable accumulation of succinate, propionate, butyrate, and formate was observed across all mineral enrichments. Quantifiable amounts of succinate (1.2–2.8 mM) were only observed in higher dilutions of ferrihydrite (10^−4^) and goethite (10^−3^) with only trace amounts of observed in hematite enrichments. Accumulation of propionate was observed across all Fe minerals, although considerably higher levels were seen in the ferrihydrite (up to 8.4 mM) and goethite (5.1 mM) enrichments as compared with hematite (trace to 0.5 mM). Up to 2.0 mM butyrate accumulated in the ferrihydrite 10^−2^ dilution with only trace amounts of butyrate detected in the goethite enrichments and none detected with hematite. Finally, as with succinate, quantifiable levels of formate (2.8 mM) were only observed in the higher dilutions of ferrihydrite (10^−4^), with trace amounts detected in all other dilutions of hematite, goethite and ferrihydrite (except 10^−1^ and 10^−3^).

Organic acid products were not detected in any of the acetate enrichments.

While it is difficult to link these results directly with the activities of the enriched phylotypes, the observed variability in the accumulation of fermentation and other metabolic products indicates distinct shifts in community metabolism that is undoubtedly related to both the iron mineralogy and initial dilution of the inoculum – these differences are currently under investigation.
